# Does very high alpha-fetoprotein affect very early hepatocellular carcinoma receiving hepatectomy?

**DOI:** 10.1007/s00423-025-03675-y

**Published:** 2025-04-09

**Authors:** Hong-Shiue Chou, Chen-Fang Lee, Hao-Chien Hung, Yin Lai, Jin-Chiao Lee, Yu-Chao Wang, Chih-Hsien Cheng, Tsung-Han Wu, Ting-Jung Wu, Kun-Ming Chan, Wei-Chen Lee

**Affiliations:** 1 Division of Liver and Transplantation Surgery, Chang-Gung Memorial Hospital, Taoyuan, Taiwan; 2 Chang-Gung University College of Medicine, Taoyuan, Taiwan

**Keywords:** Hepatocellular carcinoma (HCC), Alpha-fetoprotein (AFP), Partial liver resection, Disease-free survival (DFS), Overall survival (OS)

## Abstract

**Background:**

Following liver resection (LR), recurrence is critical to the prognosis of hepatocellular carcinoma (HCC). A higher level of alpha-fetoprotein (AFP) is typically associated with poor prognosis and recurrence concerns. Specifically, we attempted to determine whether high AFP (> 1,000ng/ml) and other potentially relevant factors affect survivals of patients with BCLC stage 0 HCC after LR.

**Methods:**

This retrospective study focused on 223 patients who received LR for stage 0 HCC of BCLC between 2004 and 2012. In patients with a low AFP (n = 200) and a high AFP (n = 23), we conducted chi-squares, independent t-test, Cox regression, and Kaplan–Meier survival analyses to investigate the relationship between clinicopathologic variables and outcomes.

**Results:**

The long-term disease-free survival (DFS) (*p* = 0.799) and the overall survival (OS) (*p* = 0.942) between the low and high AFP groups were comparable. The two groups' clinicopathologic features—tumor size, presence of a tumor capsule, cirrhosis, histology activity index (HAI), and microvascular invasion—appear to be similar. Additionally, we observed significant associations between HCC recurrence and ICG R15, HAI score, and cirrhosis, but not AFP.

**Conclusions:**

In stage 0 HCC, the consideration of curative-intent therapy in these patients should begin as soon as possible, irrespective of AFP levels.

**Supplementary Information:**

The online version contains supplementary material available at 10.1007/s00423-025-03675-y.

## Introduction

Globally, hepatocellular carcinoma (HCC) is a prevalent and deadly illness [1]. The high prevalence of hepatitis B and C infections in Asia is contributing to the high incidence of this malignancy [2].

Alpha-fetoprotein (AFP), the most widely used HCC tumor marker, has been evidently demonstrating predictive values for HCC outcomes after curative resection [3–5]. It is well known that AFP can weaken cellular immunity by influencing T lymphocytes, dendritic cells, and natural killer cells, which causes them to lose their immune surveillance ability in the battle against cancer [6, 7]. AFP is also crucial to the growth as well as the metastasis of HCC [8]. The presence of CD133, a cancer stem cell marker, in HCC tumors is associated with elevated AFP, which suggests that CD133-positive HCC cells may be responsible for tumor progression and invasiveness [9]. In addition to encouraging growth factor expression with tumor angiogenesis [10], AFP can also increase the antiapoptotic activity of cancer cells [11, 12].

A model that includes AFP in liver transplantation for HCC improves the prediction of tumor recurrence, particularly when the serum level exceeds 1,000 ng/ml [13]. Additionally, the 1,000 ng/mL cut-off point for an extremely high serum AFP level—also referred as out-score AFP—was suggested as the limit for a single HCC undergoing LR [14]. Following LR, a dispersed recurrence is also frequently observed when the pre-operative serum AFP exceeds the threshold [15]. It would seem sense that when a small quantity of tumor secretes high levels of AFP, the prognosis would decline [16]. However, in the earlier trial, there was heterogeneity in the patient composition and tumor stage. An increasing number of patients obtain an early diagnosis. Today, once an HCC has been detected, our goal is to precisely forecast the predicted result and assist medical professionals and their patients in choosing the most appropriate course of treatment.

The purpose of this study was to investigate the potential impact of extremely elevated AFP levels on clinical outcomes in Barcelona Clinic Liver Cancer (BCLC) very early stage (stage 0) HCC patients receiving curative LR.

## Materials and methods

### Study population

In this retrospective analysis, primary LR was performed on 1518 adult patients with pathologically confirmed HCC at Linkou Chang Gung Memorial Hospital (CGMH) between May 2002 and December 2012. All enrolled patients (n = 223) consented well to the indication of LR for curative-intent purposes after excluding patients who did not belong to BCLC stage 0 (n = 1273), patients who had concurrent malignant disease (n = 8), patients who were recurrent but not primary HCC (n = 2), and patients who lacked essential information for subsequent analysis (n = 12).

A BCLC stage 0 tumor allows for curative treatment options such as surgical resection or radiofrequency ablation (RFA). Favorable long-term overall survival (OS) rates for both treatments can be achieved in stage 0 HCC [17], and surgical resection is prioritized for patients with satisfactory liver function and no significant contraindications to surgery to enhance long-term disease-free survival (DFS) in our institution. In this investigation, 200 patients will be assigned to the low-value group and the remaining 23 patients to the high-value group depending on an AFP value of 1000 ng/ml. The institutional review board (IRB, No. 201503900B0) of CGMH authorized this study.

### Data collection

According to the 2022 update BCLC staging system [18], a very early stage (stage 0) for HCC is defined by the presence of a single tumor that is 2 cm or smaller in diameter. Patients at this stage typically have well-preserved liver function, usually classified as Child–Pugh class A, and they generally exhibit a good performance status, often rated as ECOG 0–1, indicating that they are fully active or only slightly restricted in their daily activities. Prior to surgery, a number of demographic factors were recorded, including age, gender, hepatitis status, the Child-Turcotte-Pugh (CTP) classification, serum total bilirubin, serum albumin, the indocyanine green (ICG) clearance test, platelet count, and serum neutrophil-to-lymphocyte ratio (NLR). The histology activity index (HAI) score, tumor diameter, cirrhosis, microvascular invasion, presence of tumor capsule and capsular invasion, tumor histology grade, and ranges of LR and resection margin were among the surgical and pathologic parameters. Based on the most recently available data (within a week) from the surgery and one month afterward, the serum AFP values were obtained.

### Post-LR HCC surveillance

At our patient clinic, every patient received routine follow-up care following LR for HCC. The follow-up periods were 91.7 ± 43.8 months for the mean and 99.0 months for the median. Patients were checked on after discharge in the first, third, and every three months thereafter. Before every clinic visit, the sonography examination and serum AFP assays were carried out. If abnormal results from blood tests and hepatic sonography suggest a tumor recurrence, arrangements would be made for computed tomography, hepatic angiography, or magnetic resonance imaging scanning.

### Clinical outcomes measurement

Disease-free survival (DFS) was the primary outcome in the current study, with overall survival (OS) and HCC recurrence patterns serving as additional outcomes. Dates of operation to the date of the latest censored follow-up or the date of an evident tumor recurrence were utilized to calculate DFS. OS was measured from the time of hepatectomy to the date of death or last available follow-up. Patients with medically established recurring HCC will be evaluated for recurrent patterns, such as recurrent period, tumor location, and disease extension. In addition, the management of recurrent HCC is similar to the principle of taking care of primary HCC and needs collaborating with multidisciplinary teams.

### Statistical analysis

For the presentation of categorical and continuous data, respectively, numbers (%) and mean values ± standard deviations (median values) were utilized. To compare clinical parameters, Pearson's chi-square test, the independent T-test, and the nonparametric approach were appropriately applied. In order to determine the impact of each parameter on the time-to-event result concerning DFS, the Cox proportional hazard risk model was selected, and a logistic regression analysis was used to determine the correlation between various hazards. All possible parameters were included in the univariate (UV) analysis and only factors with a p-value < 0.100 were later included in the multivariate (MV) analysis. Patient survival between groups was examined using the Kaplan–Meier method with a log-rank test. The predictive value of a clinical factor was uncovered using a receiver operating characteristic curve (ROC), and the Youden index was used to determine the best cut-off point for determining predictive accuracy. IBM SPSS® version 24.0 (SPSS Incorporation, Chicago, IL, USA) was the software used for all statistical analyses, and two-tailed p-values of less than 0.05 were regarded as statistically significant.

## Results

### Characteristics between the high and the low AFP groups

**Fig. 1:**
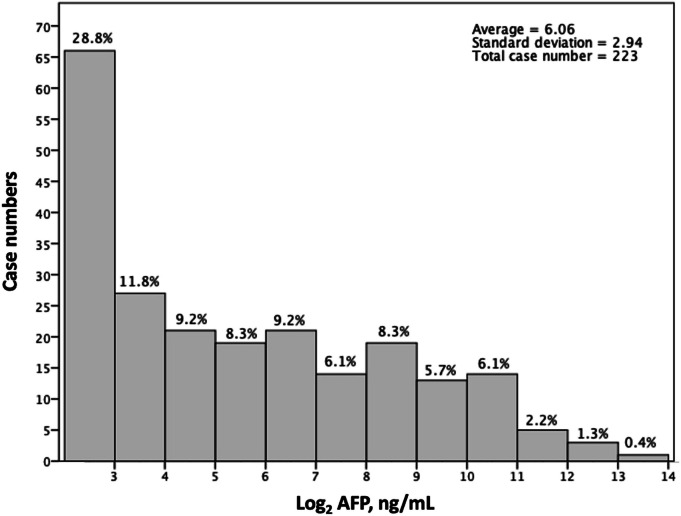
A Semi-logarithmic histogram of the case distribution over the AFP values (with Log_2_ scales) in the current study

Figure 1, a semi-logarithmic histogram, shows the AFP value with Log_2_ scales across the number of cases in the current study. Following a review of the clinical data, Table 1 provides a comparison of the clinical characteristics between the low- and the high-value AFP groups. The population consists predominantly of men (n = 163, 73.1%) and those with viral hepatitis (n = 195, 87.4%). Patients in the low-value and high-value groups had mean AFP values of 108.5 ± 188.1 and 2401.2 ± 2095.3, respectively (*p* < 0.001). While the low-value group's mean age was substantially higher than the high-value group's (49.3 ± 15.4 vs. 56.3 ± 10.8; *p* = 0.043), the two groups' proportions of older individuals (≥ 65 years old) were similar (high-value group: 21.7% vs. low-value group: 23.0%; *p* = 0.892). Furthermore, there appeared to be no difference between the two groups in terms of gender, CTP scores, and test results like NLR, platelet count, serum albumin level, and Indocyanine green retention rate at 15 min (ICG-R15). According to surgical and pathological data, major hepatectomy was performed on 57 patients (25.6%), with a comparable distribution in the two groups (high-value group: n = 6, 26.1% vs. low-value group: n = 51, 25.5%; *p* = 0.951). As predicted, there was no difference between the two groups' maximum tumor size (*p* = 0.753) or surgical resection margin (*p* = 0.789). Regarding the specifics of the tumor pathology, there was no discernible variation in the percentage of fatty change (*p* = 0.852), cirrhosis (*p* = 0.858), tumor capsular invasion (*p* = 0.664), microvascular invasion (*p* = 0.845), histology grade (*p* = 0.688), or HAI scores (*p* = 0.526).

**Table 1:** A comparative study of clinicopathologic factors in small HCC patients receiving LR according to high and low preoperative serum AFP levels

General information	AFP<1000ng/mL, n=200	AFP≥1000ng/mL, n=23	*p*-value
Age, year-old	56.3±10.8 (57.3)	49.3±15.4 (44.7)	0.043
Age ≥ 65-year-old	46 (23.0%)	5 (21.7%)	0.892
Gender, male	148 (74.0%)	15 (65.2%)	0.368
Viral hepatitis			0.415
None	27 (13.5%)	1 (4.3%)	
Chronic HBV infection	105 (52.5%)	14 (60.9%)	
Chronic HCV infection	54 (27.0%)	5 (21.7%)	
Co-infection of HBV and HCV	14 (7.0%)	3 (13.0%)	
Child-Pugh score	5.1±0.3 (5)	5.0±0.0 (5)	0.321
NLR	2.3±2.3 (1.6)	1.6±0.7 (1.7)	0.288
AFP, ng/mL	108.5±188.1 (22.1)	2401.2±2095.3 (1764.2)	<0.001
PLT, x1000/uL	157.9±61.2 (157.5)	166.5±56.9 (169.5)	0.528
ICG-R15, %	8.6±7.5 (7.0)	8.6±13.0 (5.8)	0.967
Total bilirubin, mg/dL	0.9±0.1 (0.7)	0.7±0.2 (0.6)	0.555
ALB, g/dL	4.2±0.4 (4.3)	4.3±0.4 (4.3)	0.619
Information regarding surgery and pathology			
Major hepatectomy	51 (25.5%)	6 (26.1%)	0.951
Tumor size, cm (maximum)	1.7±0.8 (1.6)	1.6±0.3 (1.6)	0.753
Resected margin, mm	7.4±6.6 (6.0)	7.0±6.0 (6.0)	0.789
Fatty change			0.852
No	57 (28.5%)	6 (26.1%)	
<33%	115 (57.5%)	15 (65.2%)	
33–66%	26 (13.0%)	2 (8.7%)	
>66%	2 (1.0%)	0 (0.0%)	
Cirrhosis	114 (58.5%)	13 (56.5%)	0.858
Microvascular invasion	17 (8.5%)	1(4.3%)	0.845
Tumor capsulation	154 (77.0%)	21 (91.4%)	0.114
Capsule invasion^b^	113 (73.3%)	18 (85.7)	0.664
Histology grade			0.688
1	21 (10.5%)	1 (4.3%)	
2	118 (59.0%)	15 (65.2%)	
3	57 (28.5%)	6 (26.1%)	
4	4 (2.0%)	1 (4.3%)	
HAI, necro-inflammatory score	4.3±2.0 (4)	4.0±2.1 (4)	0.526

^a^Abbreviation: LR liver resection, HCC hepatocellular carcinoma, AFP alpha-fetoprotein, HBV hepatitis B virus, HCV hepatitis C virus, NLR neutrophil-to-lymphocyte ratio, PLT platelet, ICG-R15 the indocyanine green retention rate at 15 min, HAI histology activity index

^b^Only calculated in patients with tumor capsule (n=165)

### Clinical outcomes between the low-value and the high-value AFP groups

**Fig. 2:**
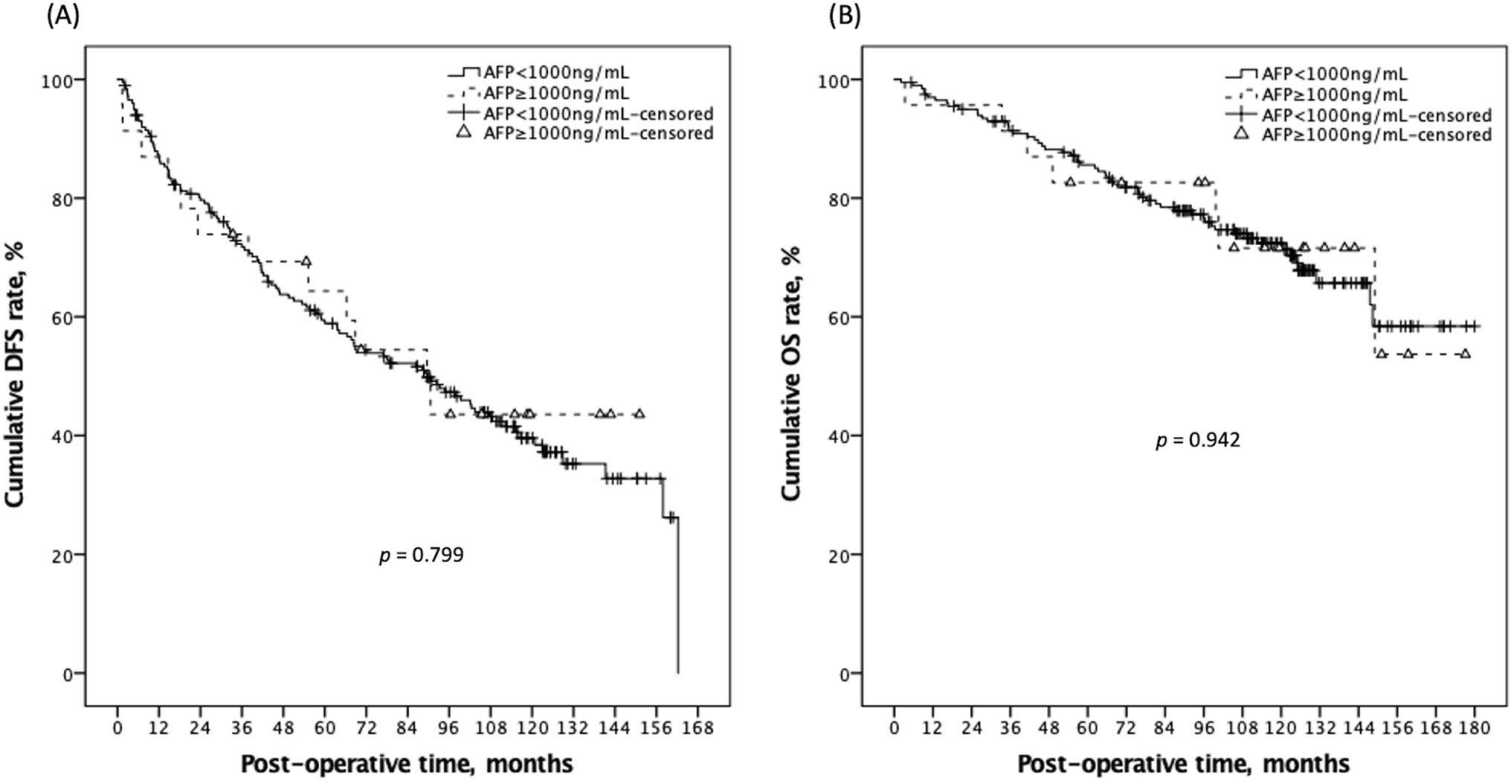
Kaplan–Meier plot of DFS and OS according to dichotomized low and high AFP groups with the cut-off value of 1000ng/ml. There was no significant survival difference on DFS (A; *p* = 0.799) and OS (B; *p* = 0.942) between the two groups. DFS, disease-free survival; OS, overall survival; AFP, alpha fetoprotein

**Fig. 3:**
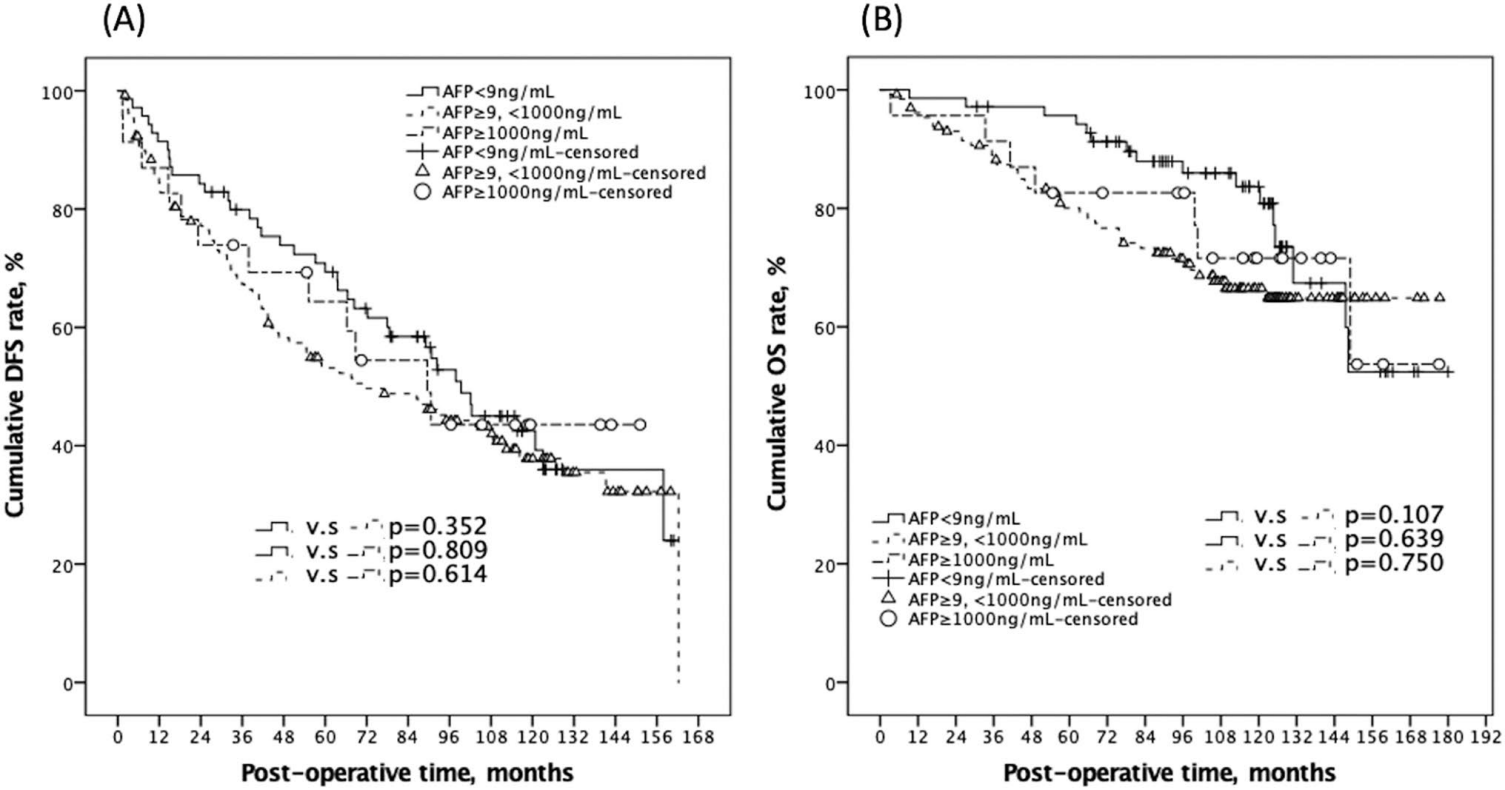
It illustrates the stratification of enrolled patients into three distinct groups based on the addition of the normal reference value of pre-operative serum AFP at 9 ng/ml to the initial cut-off point of 1000 ng/ml. This categorization allowed for a more nuanced analysis of DFS and OS outcomes. The Kaplan–Meier plots generated for each group revealed that the DFS (**A**) and OS (**B**) curves were statistically comparable across all groups, with p-values exceeding 0.05, indicating no significant differences in survival outcomes among the stratified AFP values. AFP, alpha fetoprotein; DFS, disease-free survival; OS, overall survival

Following LR, the two groups' serum AFP values decreased to similar levels in the first post-operative month (*p* = 0.325), as well as comparable oncological results were also reported (Table 2). There were 115 (57.5%) and 12 (52.2%) cumulative disease recurrences in the low-value and high-value groups, respectively, over a median follow-up duration of 99.0 months (*p* = 0.625). Patients in the low-value group had 1-, 3-, and 5-year DFS rates of 86.8%, 71.8%, and 59.5%, respectively, while those in the high-value group had 87.0%, 73.9%, and 64.3% (*p* = 0.799; Fig. 2). Further, for patients in the low-value and the high-value group, the 1-, 3-, and 5-year OS rates were 97.0%, 91.4%, 85.6%, and 95.7%, 91.3%, 82.6%, respectively (*p* = 0.942; Fig. 2). To enhance our comprehension of the potential impact of distinct AFPs on the survival rates, we categorized them into three groups by appending the normal reference value (9 ng/ml) to the initial cut-off point of 1000 ng/ml. The findings demonstrated that similar DFS and OS curves were observed in patients with stratified AFP values in stage 0 HCC (all *p* > 0.05; Fig. 3). These findings suggest that the incorporation of the normal and extremely high reference values of pre-operative AFP does not adversely affect patient outcomes. Supplemental Fig. 1 displayed a more detailed KM survival curve among different AFP values (with Log_2_ scales) with corresponding DFS and OS, and there was no significant p-value between any two groups. Notably, four of our cases had pre-LR AFP data greater than two to the 13th power, and three of them continued to be cancer-free until the study's observation period ended.

**Table 2:** Clinical outcomes and recurrent patterns of small HCC patients after LR according to high and low preoperative serum AFP levels

Clinical outcomes	AFP<1000 ng/mL, n=200	AFP≥1000 ng/mL, n=23	*p*-value
Post-LR HCC DFS rate, at 1-, 3-, 5-year, %	86.8%/71.8%/59.5%	87.0%/73.9%/64.3%	0.799
Cumulative recurrence, number	115 (57.5%)	12 (52.2%)	0.625
Post-LR OS rate, at 1-, 3-, 5-year, %	97.0%/91.4%/85.6%	95.7%/91.3%/82.6%	0.942
Cumulative death, number	57 (28.5%)	7 (30.4%)	0.846
POM 1 Serum AFP, ng/mL	17.6±32.7 (7.9)	23.4±17.5 (18.6)	0.325
Condition at the time of recurrence^b^	AFP<1000 ng/mL, n=115	AFP≥1000 ng/mL, n=12	*p*-value
Recurrent period since surgery, months	47.4±38.8	39.5±33.4	0.498
Extrahepatic recurrence, yes	8 (7.0%)	1 (8.3%)	0.860
Intrahepatic recurrence, multiple	55 (47.8%)	7 (58.3%)	0.488
Recurrence from resection margin, <10mm	9 (7.8%)	1 (16.1%)	0.300
Recurrence with DBMC, yes	23 (20.0%)	4 (33.3%)	0.283

^a^Abbreviation: LR liver resection, HCC hepatocellular carcinoma, AFP alpha-fetoprotein, DFS disease-free survival, OS overall survival, POM post-operative month, DBMC disease beyond Milan criteria

^b^Only calculated in 127 patients with HCC recurrence

### Recurrent patterns

Staging should commence with looking for evidence of extrahepatic disease (presented in nine patients in the current study, eight (7.0%) in the low-value group and one (8.3%) in the high-value group; *p* = 0.860) after the diagnosis of HCC recurrence (n = 127) has been established. All patients had Intrahepatic recurrence. A multiplicity of tumor numbers was presented in nearly half of patients (n = 62, 48.8%) irrespective of pre-LR AFP levels (high-value group: n = 7, 58.3% vs. low-value group: n = 55, 47.8%; *p* = 0.488). Since no formal staging schema has been defined yet relative to recurrent HCC, the Milan criteria is frequently used to help predict outcomes and guide decision-making in the case of recurrent HCC, especially when a liver transplant is a therapeutic option worth considering. Of all patients with HCC recurrence, 27 (21.3%) had disease beyond Milan criteria (DBMC). There was no statistically significant difference between the two groups (high-value group: n = 4, 33.3% vs. low-value group: n = 23, 20.0%; *p* = 0.283).

### Prognostic factors affecting DFS

Table 3 demonstrated that most clinicopathological factors, including age, gender, albumin, Child–Pugh score, AFP, tumor size, hepatectomy extension, tumor resection margin, tumor capsulation, and the presence of microvascular invasion, capsular invasion, histology grade, and fatty change percentage, were not potential predictors (all *p* > 0.100) in the UV cox regression analysis to predict post-LR HCC recurrence, but ICG-R15 (HR: 1.05, 95% CI: 1.03–1.06, *p* < 0.001), cirrhosis (HR: 1.90, 95% CI: 1.31–2.76,* p* = 0.001), chronic hepatitis C virus (HCV) infection (HR: 1.74, 95% CI: 0.95–3.17, *p* = 0.072), and HAI scores (HR: 1.17, 95% CI: 1.08–1.27,* p* < 0.001). Substantially, ICG-R15 (HR: 1.03, 95% CI: 1.01–1.05, *p* = 0.004), cirrhosis (HR: 1.54, 95% CI: 1.04–2.28, *p* = 0.030), and HAI scores (HR: 1.11, 95% CI: 1.02–1.21, *p* = 0.015) were found to be associated significantly with post-LR HCC recurrence in the MV cox regression model.

**Table 3:** Uni-/multivariate analyses for clinicopathological factors to predict HCC recurrence in HCC patients after LR using Cox regression

Parameters	Univariate^b^			Multivariate		
	HR	95%CI	*p*-value	HR	95%CI	*p*-value
ICG R15, %	1.05	1.03–1.06	< 0.001	1.03	1.01–1.05	0.004
Cirrhosis presence v.s absence	1.90	1.31–2.76	0.001	1.54	1.04–2.28	0.030
Chronic HCV infection v.s none	1.74	0.95–3.17	0.072			
HAI, necro-inflammatory score	1.17	1.08–1.27	< 0.001	1.11	1.02–1.21	0.015

^a^Abbreviation: LR, liver resection; HCC, hepatocellular carcinoma; HCV, hepatitis C virus; ICG-R15, the indocyanine green retention rate at 15 min; HAI, histology activity index

^b^Following pre-operative factors (categorized by cut-off values) were calculated in the UV:  Age, gender, viral hepatitis, pre-LR serum NLR, total bilirubin, albumin, AFP (1000ng/mL), Child-Pugh score, tumor size, resection margin, hepatectomy extension, histology grade, capsulation, capsule invasion, microvascular invasion, HAI score, cirrhosis, fatty change percentage; only significant results (*p*-value < 0.100) were shown in this table and entering the multivariate analysis

**Fig. 4:**
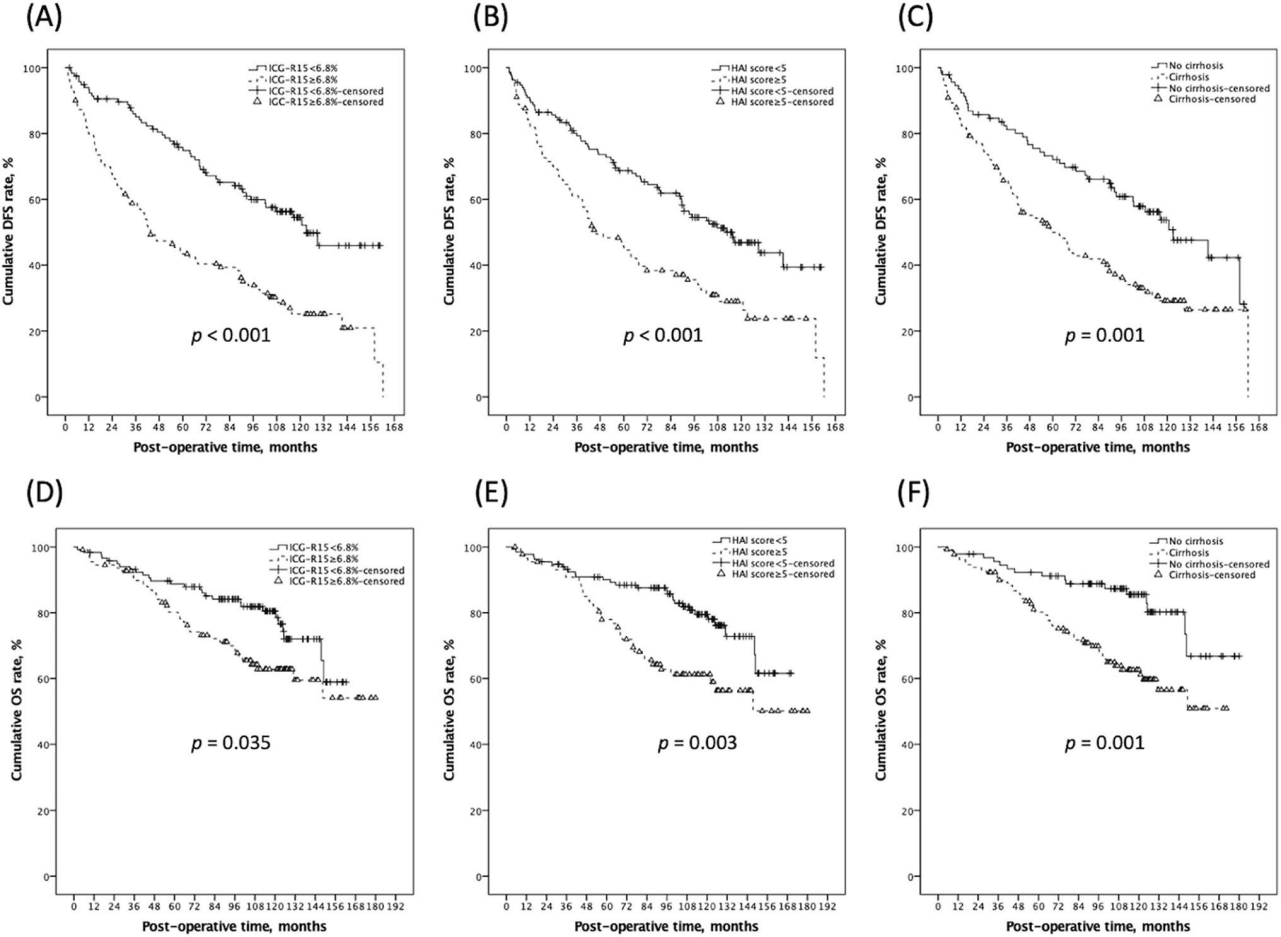
The noticeable impact that independent risk factors possessed on DFS and OS. Patients with greater ICG-R15 (A; *p* < 0.001), higher HAI score (B; *p* < 0.001), and cirrhosis (C; *p* = 0.001) had correspondingly lower DFS rates. As expected, there was a negative correlation between the OS rates and a higher ICG-R15 (A; *p* = 0.035), a higher HAI score (B; *p* = 0.003), and cirrhosis (C; *p* = 0.001). DFS, disease-free survival; OS, overall survival; ICG-R15, Indocyanine green retention rate at 15 min; HAI, histology activity index

For better clinical application, ROC analyses (Supplemental Fig. 2) were performed to quantify ICG-R15 and HAI scores in order to decide optimal cut-off thresholds to predict DFS. The AUROC of ICG-R15 and HAI scores in predicting HCC recurrence was 0.669 (95% CI: 0.599–0.740) and 0.629 (95% CI: 0.556–0.703). By optimal division, the correlated sensitivity, specificity, positive predictive value (PPV), negative predictive value (NPV), and accuracy were 60.6%, 68.8%, 83.0%, 72.0%, and 64.1% for ICG-R15 divided by 6.8% and 48.8%, 71.9%, 69.7%, 51.5%, and 58.4% for HAI scores divided by 5, in anticipating HCC recurrence (Supplemental Table 1). Figure 4 exhibited the significant influence these independent risk factors had on both DFS and OS. The corresponding 1-, 3-, and 5-year post-LR DFS rates were 94.0%, 85.0%, and 75.9% among patients with an ICG-R15 < 6.8% and 80.4%, 56.3%, and 42.2% among those with an ICG-R15 ≥ 6.8% (log-rank test *p* < 0.001). The ICG-R-15 is the one and only pre-operative risk that has been found in the current study. As anticipated, OS rates were negatively impacted by a higher ICG-R15 level (log-rank test* p* = 0.035).

## Discussion

Following a hepatectomy, the distribution of recurrence was relatively lower in the early years and did not show a slow drop with time. The long-term outcomes and recurrent patterns between the low- and high-value groups were similar, according to our findings. With a discrimination of 24 months for recurrent time, among the 127 cases with post-hepatectomy HCC recurrence, 80 (63.0%) of them were late recurrence. In our study, we found that several cirrhosis-associated factors (ICG R-15, cirrhosis, and HAI scores) were indicative for post-LR HCC recurrence. Similar conclusion has been made on previous studies that HCC late recurrence has an association with cirrhosis [19, 20]. On the other hand, patients with cirrhosis often present with multifocal disease or microvascular invasion, resulting in a higher likelihood of residual tumor cells remaining after resection. This residual disease can contribute to early recurrence [21]. Besides, cirrhosis can possibly influence early recurrence in HCC patients following curative partial liver resection through mechanisms such as creating a pro-tumor fibrotic microenvironment that supports tumor survival, impairing hepatic regeneration, altering vascular dynamics due to portal hypertension, and promoting immune dysfunction and inflammation, all of which may facilitate tumor regrowth and recurrence [22–25].

Background liver cirrhosis serves as an important hepatocarcinogenetic factor for post-hepatectomy recurrence, and further compromises outcomes [20, 26]. Before choosing a course of treatment, patients with varying degrees of liver cirrhosis should have their cirrhosis severity assessed. No conclusive recommendations have been given about the management for solitary and small HCC with cirrhosis. It is believed that liver transplantation can treat both the background cirrhosis and the tumor [27]. The severity of liver cirrhosis should be assessed before surgery and may help to personalize therapeutic approaches [28].

Four necroinflammation grading and one fibrosis staging categories made up the HAI system. These are significant tumorigenic characteristics of the residual liver parenchyma following the removal of the primary tumor for late recurrence [29, 30]. Patients with multicentric HCCs had considerably greater rates of periportal necrosis, focal necrosis, piecemeal necrosis, and intralobular degeneration than those patients without them. A close relationship between multicentric carcinogenesis and histologically evident hepatocytic necrosis was also observed [31].

A similar article by Sasaki et al. [32] highlights the significance of LR in resectable HCC with implications of extremely high preoperative AFP levels (> 4,000 ng/mL), demonstrating that despite extremely elevated AFP levels, surgical intervention can lead to long-term favorable prognoses. However, it is concerning that the study population exhibited high heterogeneity, which may impact the generalizability of the findings. We conduct a supplemental comprehensive analysis using Kaplan–Meier survival curves, which included varying AFP values presented on a Log2 scale. Our results indicated no significant p-value between the groups analyzed, suggesting that the differences in survival outcomes were not clinically meaningful. Notably, one of the values is 4096 (which is 212), which is very close to the proposed cutoff of 4000. Given this proximity, we believe that our findings emphasize the necessity of considering curative resection as a vital therapeutic strategy in very early stage HCC treatment protocols.

The percentage of people with extremely high AFP is growing and currently accounts for 10% of cases of BCLC stage 0 HCC, as screening awareness and diagnostic capabilities increase [33]. Our findings further suggest that background liver parenchyma, not tumor derived variables like AFP, is linked to HCC recurrence in very early stage HCC. High AFP concentrations have been linked to large tumor size and low tumor cellular differentiation [34]—two characteristics that are directly associated to microvascular invasion (MVI), refers to the penetration of cancer cells into the small blood vessels located in the liver tissue adjacent to the tumor [35, 36]. Currently, MVI is one of the most significant clinical markers for anticipating the recurrence of HCC [37]. The tumor is confined and lacks intrahepatic metastases when it is discovered and treated at an early stage; this highlights the tumor's amenability to curative-intent therapy, even though it is accompanied by a very high level of AFP. This can be deduced from the current study's comparatively low MVI ratio. A propensity-matching study also revealed that there was no proof of a relationship between serum AFP levels and HCC metastases in small HCC [38].

Nonetheless, since the very early stage of the patient population were the focus of this investigation, the findings demonstrated that cellular differentiation and AFP are not always directly correlated with tumor size. In turn, patients of the BCLC stage 0 HCC with tumor recurrence represent a high-involvement situation in terms of tumor morphology, biologic behavior, and pro-tumorigenic ability of the background liver parenchyma.

## Limitation

This study's primary strength is that it minimizes study population heterogeneity by focusing on HCC at an early stage receiving LR and includes essential pathology information and long-term follow-up. There were limitations to this single-institution analysis because of its retrospective design and relatively small sample size. Additionally, this analysis did not assess other tumor markers such as Lens culinaris agglutinin reactive AFP (AFP-L3), PIVKA-II, alpha-l-fucosidase (AFU), and prothrombin des-r-carboxy prothrombin (DCP). Furthermore, the next phases in size and number growth of these highly AFP-producing HCCs are unknown to us. The authors hope that the preciousness of our research can be seen and we believe that our qualitative survival results that provide deeper insights into the implications of high AFP levels in small HCCs, which could complement quantitative findings. These results should encourage specific areas for future research that could validate our findings with larger and prospective samples.

## Conclusions

Conclusively, very high AFP could not distinguish between distinct recurring risks in BCLC stage 0 patients; therefore, it is not a suitable criterion for selecting candidates or a predictor for these specific stage patients undergoing LR to cure HCC.

## Supplementary Information

Below is the link to the electronic supplementary material.

Supplementary file1 Figure 1. More in-depth Kaplan-Meier survival curves with associated DFS (A) and OS (B) were shown for varying AFP values (using Log2 scales), and no significant p-value was found between any of the groups. DFS, disease-free survival; OS, overall survival (JPG 218 KB)

Supplementary file2 Figure 2. The ICG-R15 and HAI scores were quantified using ROC analysis to determine the best threshold for DFS prediction. With the best cut-off values, ICG-R15 and HAI scores had an AUROC of 0.650 (95% CI: 0.578-0.722) and 0.611 (95% CI: 0.537-0.686) in predicting HCC recurrence, respectively. ICG-R15, Indocyanine green retention rate at 15 minutes; HAI, histology activity index; ROC, receiver operating characteristic; DFS, disease-free survival; AUROC, area under ROC (JPG 144 KB)

Supplementary file3 (DOCX 16 KB)

## Data Availability

No datasets were generated or analysed during the current study.
