# Supplementary figures and images for: Does very high alpha-fetoprotein affect very early hepatocellular carcinoma receiving hepatectomy?
Source: Langenbecks Arch Surg. 2025 Apr 9;410(1):124. doi: 10.1007/s00423-025-03675-y (PMC11982121; doi:10.1007/s00423-025-03675-y)

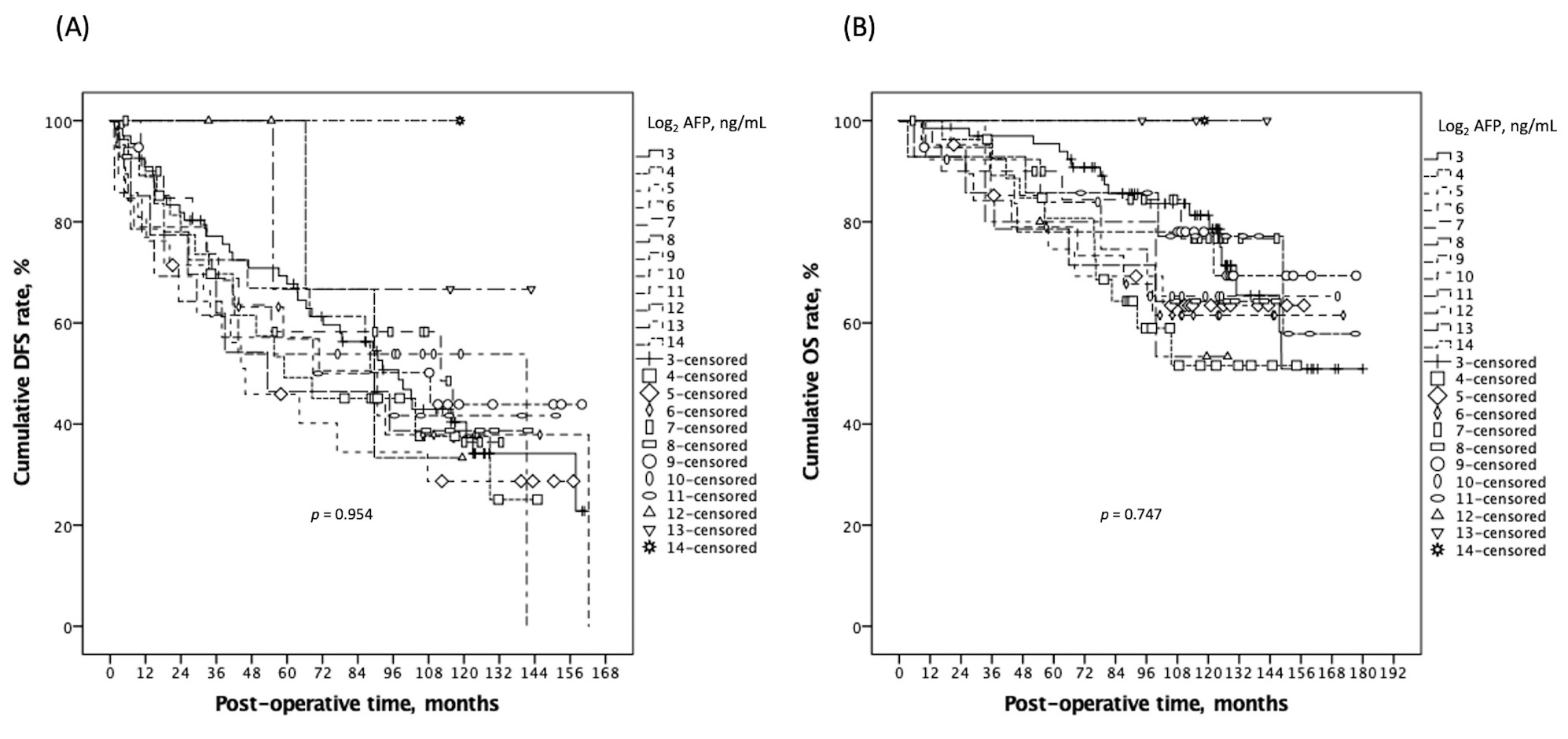

Supplement: Supplementary file 1 — Supplementary file1 Figure 1. More in-depth Kaplan-Meier survival curves with associated DFS (A) and OS (B) were shown for varying AFP values (using Log2 scales), and no significant p-value was found between any of the groups. DFS, disease-free survival; OS, overall survival (JPG 218 KB) [file 423_2025_3675_MOESM1_ESM.jpg]

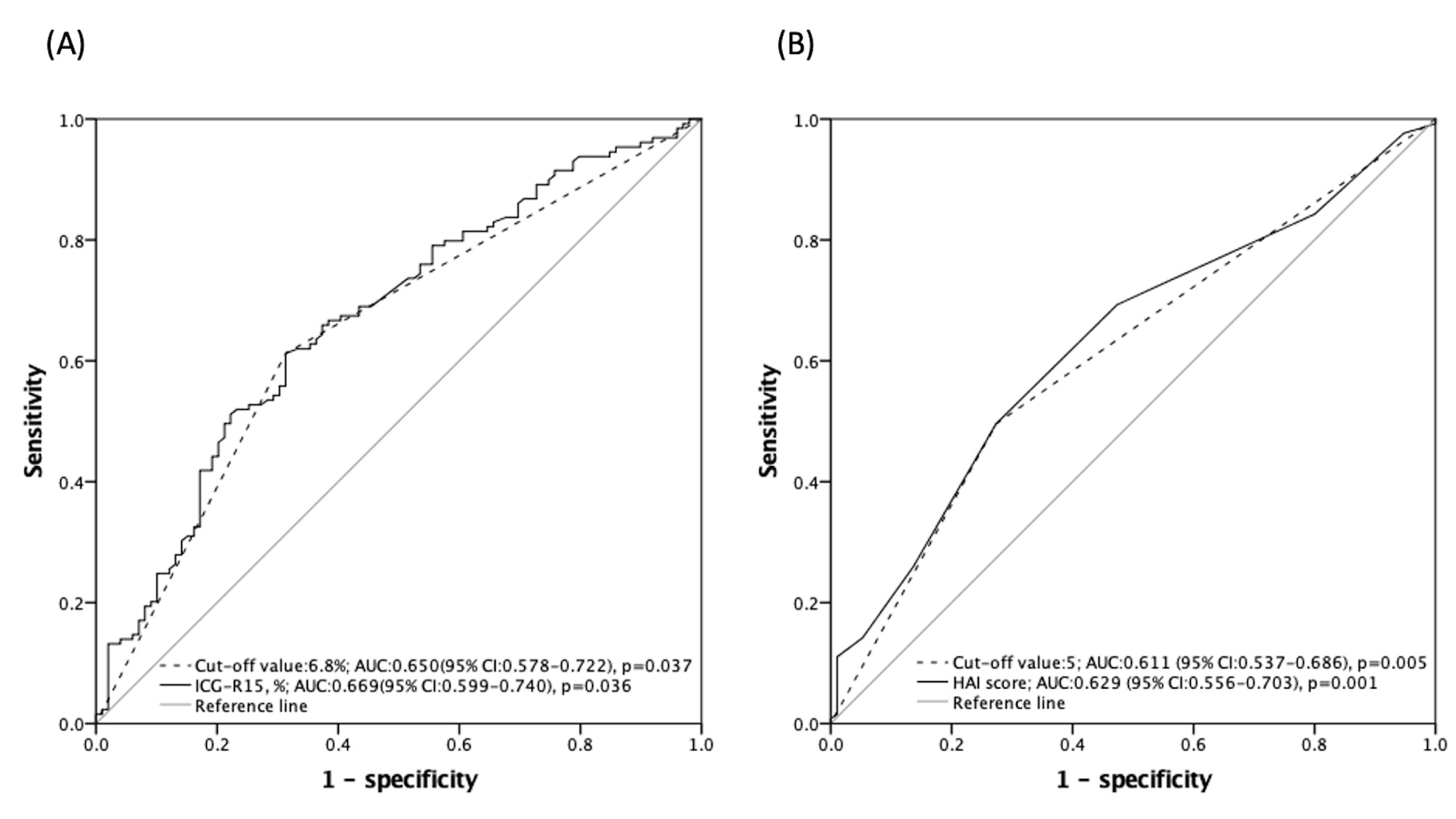

Supplement: Supplementary file 2 — Supplementary file2 Figure 2. The ICG-R15 and HAI scores were quantified using ROC analysis to determine the best threshold for DFS prediction. With the best cut-off values, ICG-R15 and HAI scores had an AUROC of 0.650 (95% CI: 0.578-0.722) and 0.611 (95% CI: 0.537-0.686) in predicting HCC recurrence, respectively. ICG-R15, Indocyanine green retention rate at 15 minutes; HAI, histology activity index; ROC, receiver operating characteristic; DFS, disease-free survival; AUROC, area under ROC (JPG 144 KB) [file 423_2025_3675_MOESM2_ESM.jpg]
