# Supplementary material for: Does very high alpha-fetoprotein affect very early hepatocellular carcinoma receiving hepatectomy?
Source: Langenbecks Arch Surg. 2025 Apr 9;410(1):124. doi: 10.1007/s00423-025-03675-y (PMC11982121; doi:10.1007/s00423-025-03675-y)
Supplement: Supplementary file 3 — Supplementary file3 (DOCX 16 KB) [file 423_2025_3675_MOESM3_ESM.docx]

| Supplemental Table 4. Accuracy in predicting post-PLR HCC recurrence and for very early stage HCC patients | | | | | | | |
| --- | --- | --- | --- | --- | --- | --- | --- |
| Predicted factor | AUROC  (95% CI) | Optimal  cut-point | Sensitivity | Specificity | PPV | NPV | Accuracy |
| ICG-R15, %  HAI score | 0.669 (0.599-0.740)  0.629 (0.556-0.703) | 6.8  5 | 60.6%  48.8% | 68.8%  71.9% | 83.0%  69.7% | 72.0%  51.5% | 64.1%  58.4% |
| 1. Abbreviation: PLR, primary liver resection; HCC, hepatocellular carcinoma; AFP, alpha-fetoprotein; ICG-R15, the indocyanine green retention rate at 15 min; HAI, hepatitis activity index; AUROC, area under the receiver operating characteristics curve; PPV, positive predictive value; NPV, negative predictive value. | | | | | | | |
